# Erratum: Airway T cells protect against RSV infection in the absence of antibody

**DOI:** 10.1038/mi.2017.79

**Published:** 2017-08-30

**Authors:** E Kinnear, L Lambert, J U McDonald, H M Cheeseman, L J Caproni, J S Tregoning

**Keywords:** T cells, Cell death and immune response, Viral infection, Respiratory tract diseases

**Correction to:** *Mucosal Immunology* advance online publication 24 May 2017; doi: 10.1038/mi.2017.46

This article was originally published under NPG's License to Publish, but has now been made available under a [CC BY 4.0] license. The PDF and HTML versions of the paper have been modified accordingly.

